# DPCT: A Dynamic Method for Detecting Protein Complexes From TAP-Aware Weighted PPI Network

**DOI:** 10.3389/fgene.2020.00567

**Published:** 2020-06-26

**Authors:** Ali SabziNezhad, Saeed Jalili

**Affiliations:** Computer Engineering Department, Tarbiat Modares University, Tehran, Iran

**Keywords:** protein complex, PPI network, TAP data, memetic algorithm, biclustering

## Abstract

Detecting protein complexes from the Protein-Protein interaction network (PPI) is the essence of discovering the rules of the cellular world. There is a large amount of PPI data available, generated from high throughput experimental data. The enormous size of the data persuaded us to use computational methods instead of experimental methods to detect protein complexes. In past years, many researchers presented their algorithms to detect protein complexes. Most of the presented algorithms use current static PPI networks. New researches proved the dynamicity of cellular systems, and so, the PPI is not static over time. In this paper, we introduce DPCT to detect protein complexes from dynamic PPI networks. In the proposed method, TAP and GO data are used to make a weighted PPI network and to reduce the noise of PPI. Gene expression data are also used to make dynamic subnetworks from PPI. A memetic algorithm is used to bicluster gene expression data and to create a dynamic subnetwork for each bicluster. Experimental results show that DPCT can detect protein complexes with better correctness than state-of-the-art detection algorithms. The source code and datasets of DPCT used can be found at https://github.com/alisn72/DPCT.

## Introduction

Protein complexes are modules made up of some proteins, which become a group at a specific time and situation, to become a functional part of a biological process (Gavin et al., 2006). Research about protein complexes can help in obtaining a better understanding of cellular systems. The importance of investigating protein complexes has caused many researchers around the world to create large amounts of experimental data such as protein-protein interaction (PPI) data and gene expression data (GE) (Bader and Hogue, 2002). Considering the enormous size of experimental data and the cost of experimental methods, it is necessary to define computational methods to process these data and to detect protein complexes (Enright et al., 2002). Many methods have therefore been proposed to detect protein complexes from PPI networks (Li et al., 2010).

A basic method to detect protein complexes from the PPI network is clustering. MCL (Enright et al., 2002) proposes to detect protein complexes by clustering the PPI network using random walking. MCL is very useful and scalable but it cannot detect overlapping protein complexes. In recent years, Ou-Yang et al. (2016a) introduced TINCD which consists of two layers. In the first layer, adjacency matrices are created for both PPI and TAP data and ensemble learning is applied to detect protein complexes from each matrix. TINCD uses 11 state-of-the-art methods on the PPI network and five detection methods on TAP data to detect protein complexes. Two create similarity matrices and a scoring matrix, induced from PPI and TAP adjacency matrices, is the input to the second layer of TINCD. In the second layer, TINCD applies similarity network fusion to detect final protein complexes. PSMVC (Ou-Yang et al., 2016b) is another detection method that uses both PPI and TAP data. For each dataset, an adjacency matrix is created for specific patterns and another matrix is created for mutual patterns between PPI and TAP using learning algorithms. Finally, PSMVC detects protein complexes from these three matrices like TINCD. Ramadan et al. (2016) proposed a genetic-based method to detect protein complexes from the PPI network. In Ramadan's method, first, a clustering algorithm is applied to the PPI network and then a genetic algorithm is used to improve detected clusters by improving the cluster's correlation using Gene Ontology (GO) data.

GMFTP (Zhang et al., 2014) is a generative model with functional and topological properties. This method tries to find overlapping protein complexes using the PPI network and functional profile. GMFTP defines four scores for protein-complex affinity, complex-function preference, protein-function association, and protein-protein interaction. Based on these scores, GMFTP generates a protein-complex membership indication matrix and detects protein complexes using that matrix. Another new method to detect protein complexes is InteHC (Wu et al., 2013) which relies on integrating heterogeneous biological data to make a protein-protein interaction network. InteHC uses PPI, GO, TAP, and GE separately and defines a formulation for each data source to find if proteins p and q have interaction. In the next step, InteHC uses some known positive and negative protein-protein interactions and applies a linear support vector machine (SVM) to learn from this training data and to generate a weight for each interaction. In the final phase, InteHC applies a hierarchical clustering algorithm with three different formulations to detect protein complexes from the protein-protein interaction network. ONCQS (Zhao and Lei, 2019) uses the quotient space theory to detect protein complexes. The method makes some maximum complete subgraphs from the PPI network and detects overlapping protein complexes. ONCQS uses GO to assign a weight to each interaction of the PPI network.

Unlike previous methods that use just clustering, some detection algorithms are seed-based. ClusterONE (Nepusz et al., 2012), introduced by Nepusz et al., starts from each protein and tries to grow it up by a greedy algorithm to make a protein complex. ClusterONE attaches each neighbor protein to a preliminary complex according to its cohesiveness amount. The cohesiveness of a protein to a preliminary complex is the proportion of its intra interactions to its extra interactions, so, a higher value of cohesiveness indicates a more likely protein to attach to the preliminary complex. Finally, ClusterONE merges highly overlapping complexes to achieve the final set of protein complexes. CSeq-GO (Yu et al., 2017) detects protein complexes in three steps: making weighted PPI, feature selection, and protein complex detection. First, a gene ontology graph and amino acid frequency (topology-sequenced information) are used to make a weighted PPI network and then a protein complex detection algorithm is applied to the weighted PPI network. CSeq-GO detects protein complexes based on density, network diameter, and the included angle cosine.

Scientists have proved that we can divide proteins in a protein complex into two part called the core and attachment parts (Gavin et al., 2006). The core proteins are the main functional part of the complex and the attachment proteins act as help for the core part. In case of density, core proteins have more interaction among themselves and attachment proteins are the environmental proteins for a core. There are many methods that detect protein complexes by taking core-attachment structure into account. COACH (Wu et al., 2009) detects cores by finding dense subsets of the PPI network based on a threshold and adds attachment proteins to the detected cores. A protein is considered an attachment to a core if adding it to the core increases the weight of the total complex. CAMWI (Lakizadeh et al., 2015a), a core-attachment based algorithm, detects protein complexes in four steps. First, it chooses seeds to find cores based on a threshold; then, seeds are grown up to make cores and in the third step it adds attachment proteins to each core. Finally, it filters the results and removes highly similar detected protein complexes.

Mehranfar et al.'s method (Mehranfar et al., 2017) is similar to CAMWI and has three major steps (seed generation, core finding, and core growing) but it differs in making the weighted PPI network. Mehranfar's method uses three graphs in the GO dataset and for each graph applies (Resnik, 1995; Lin, 1998; Hwang et al., 2007) algorithms to define weight to each interaction of the PPI network. These data and other information come from comparing the structure of proteins which are inputs to a fuzzy (Zadeh, 1965; Mendel and John, 2002) function which makes the final weight of each interaction in the PPI network. Finally, the main three-step core-attachment protein complex detection method is applied to the weighted PPI network. EWCA (Wang and Caixia Wang, 2019) first uses Jaccard's coefficient similarity and a new high-order common neighborhood score to assign a weight to each interaction of the PPI network. In the next phase, EWCA starts to detect cores. Each core should have more than two proteins and all proteins should be connected to each other and have a heavier weight than other neighbors; all proteins should have high functional similarity. In the next phase, the algorithm finds potential attachment proteins and in the last step, protein complexes are formed by adding attachments to the cores.

Most protein complex detection methods use a static PPI network as their dataset but it is shown that cellular systems are dynamic in nature (Srihari and Leong, 2012), so the PPI network will change over time/conditions. Considering this, static PPI cannot represent the true nature of protein interactions across time/condition. Most of the recent methods try to take the dynamicity of cellular systems into account by creating dynamic PPI from static PPI, using time-course Gene Expression (GE) data (Hanna et al., 2015). Time-course gene expression data is a matrix where each row represents a protein and each column represents a time stamp. GE provides us with the expression level of each protein during the microarray experiment.

TSN-PCD (Li et al., 2012) makes dynamic subnetworks for each time point of gene expression data and it uses hierarchical clustering called HC-PIN to detect protein complexes from each subnetwork. After removing redundant detected protein complexes, the final result is the union of all detected complexes in each subnetwork. TS-OCD (Ou-Yang et al., 2014) divides all interactions of the PPI network into stable and temporal, where stable interactions appear in all time points of gene expression and temporal interactions appear only in parts of the time points. After constructing dynamic subnetworks, protein complex detection starts using OCD hierarchical clustering and the final result is a set of protein complexes gathered from each dynamic subnetwork.

There are some methods that use both dynamicity and core-attachment approaches to detect protein complexes. Lakizadeh et al. (2015b) introduced PCD-GED which uses a threshold to separate active and inactive proteins in gene expression data and makes dynamic PPI subnetworks. In the next step, PCD-GED chooses some proteins as seeds and grows them up in a greedy way to make cores and finally to add attachments to detected cores. DPC-NADPIN (Shen et al., 2016) used gene expression data with 36 time points and made 36 dynamic PPI subnetworks. After finding cores based on the clustering coefficient and a threshold, DPC-NADPIN tries to add attachment proteins based on the proportion of inside and outside interactions between the protein and core.

Taking the dynamicity of the cellular system into account, the quality of detection methods has increased and in recent years, using biclustering instead of clustering—another improvement in the quality of detecting protein complexes. In methods like DPC-NODPIN, the PPI network is partitioned to some dynamic subnetworks based on time points of GE data. So, we assume in each time point, only active proteins can contribute to make a protein complex. On the other hand, biclustering allows us to detect more biclusters (i.e., matrices of some proteins and some time points). Moreover, considering each bicluster, all of its proteins that are active in some time point, have a better chance to participate in forming a protein complex.

BiCAMWI (Lakizadeh and Jalili, 2016) is one of the methods that uses a genetic algorithm to detect dynamic biclusters from gene expression data. For each detected bicluster, BiCAMWI makes a dynamic subnetwork of PPI and protein complexes are detected from each subnetwork separately by applying CAMWI. The main idea of BiCAMWI is using a metaheuristic algorithm to make dynamic PPI and using biclustering instead of clustering, which makes dynamic subnetworks more accurate. After BiCAMWI, PCD-DPPI (Janani et al., 2018) was proposed and used as a shuffled frog-leaping algorithm instead of the genetic algorithm to bicluster gene expression data. The shuffled frog leaping algorithm needs less time to converge vs. a genetic algorithm. After making biclusters, PCD-DPPI makes dynamic subnetworks and finds protein complexes from them. IFPA (Lei et al., 2019) uses a nature-inspired optimization algorithm called FPA (flower pollination algorithm) to detect protein complexes. IFPA generates 12 dynamic PPI subnetworks based on GE timestamps and divides interactions to certain and not certain interactions and defines a co-essentiality value between two proteins. In IFPA, co-localization, co-annotation, and co-cluster values are also defined. The algorithm finds cores based on density and applies an improved FPA to find the attachment proteins for cores and detects final protein complexes.

In this study, we present a novel **d**ynamic method to detect **p**rotein **c**omplexes from the **T**AP-Aware weighted PPI network (DPCT) which uses a memetic metaheuristic algorithm for biclustering gene expression data, which can detect more accurate biclusters and is time efficient rather than a genetic algorithm. Using TAP data along with GO gives us a precise weighted PPI network. A post-processing step in DPCT analyses and aggregates also detects protein complexes from each dynamic PPI subnetwork and removes highly similar or redundant protein complexes.

In section Materials and Methods, we introduce datasets and benchmarks used in the evaluation of DPCT and define all phases of DPCT separately. In section Experiments and Results, we evaluate the proposed method and compare it with state-of-the-art methods. Section Analytical Discussion assesses the effect of each novelty in DPCT and we conclude the paper in section Conclusion.

## Materials and Methods

### Datasets

We use DIP and BioGrid PPI networks to measure the effectiveness of the proposed method. The DIP (Salwinski et al., 2004) PPI network consists of 21,592 interactions among 4,850, and the BioGrid (Chatr-aryamontri et al., 2013) PPI network consists of 59,748 interactions between 5,640 proteins. To make a dynamic PPI subnetwork, DIP expression data were used. In DIP expression data, 2,390 proteins are expressed in 12 time courses. To make weighted PPI, we used two separate datasets including GO and TAP. TAP data consists of two datasets that come from two experiments named LCMS and MALDI in research by Krogan et al. (2006).

To evaluate the correctness of detected protein complexes, two benchmark datasets were used. CYC2008 (Pu et al., 2008) with 408 protein complexes among 1,627 proteins, and MIPS (Mewes et al., 2004) with 313 protein complexes among 1,225 proteins.

### Method

**Figure 1:**
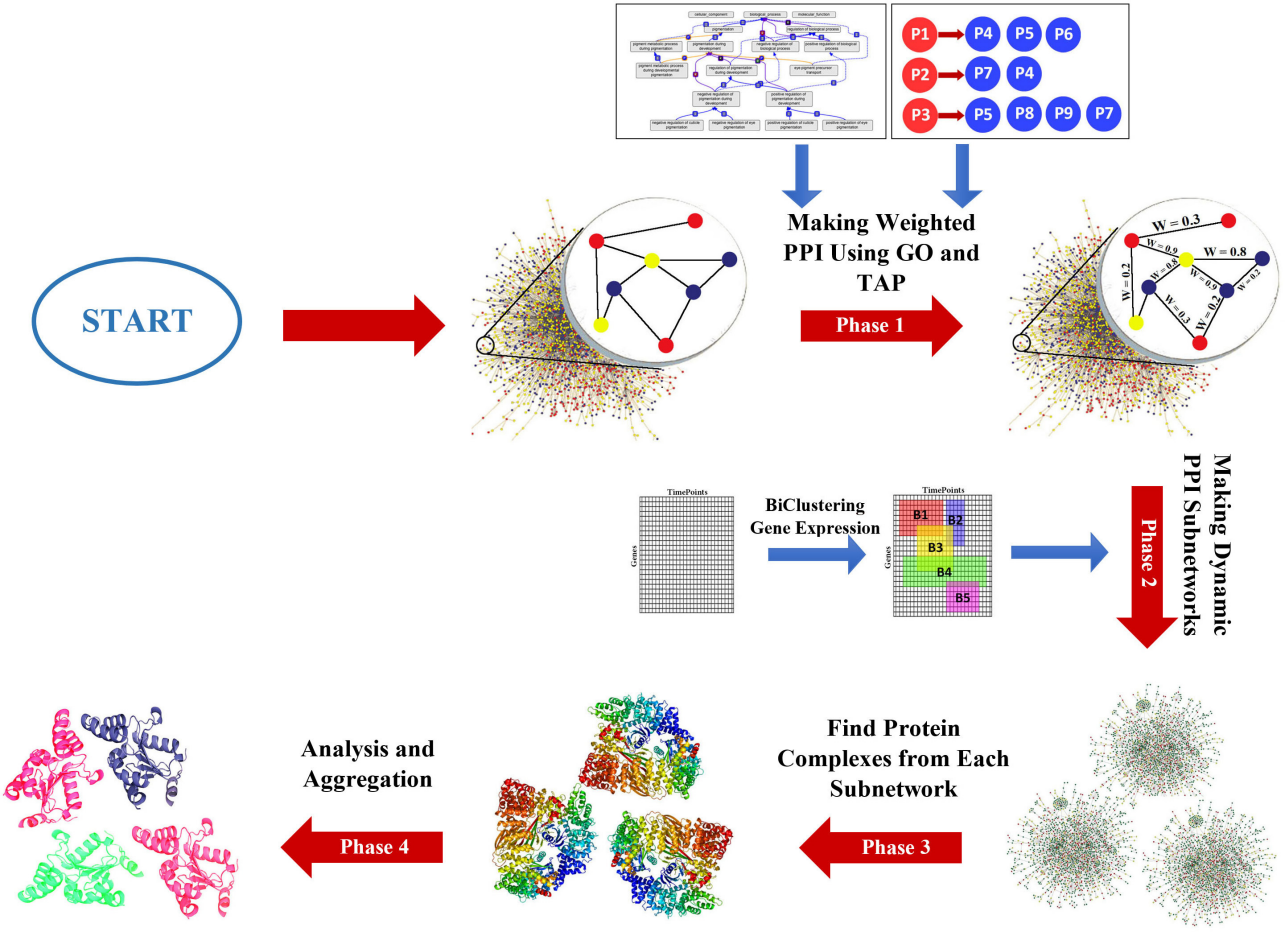
The main structure of DPCT method.

The DPCT method includes four phases as shown in Figure 1. Phase 1, assigns a weight to each interaction of the PPI network using GO and TAP datasets. The second phase generates some dynamic subnetworks from the PPI network using a memetic metaheuristic algorithm. Phase 3 detects protein complexes from each dynamic PPI subnetwork and finally the fourth phase, analyzes and aggregates detected protein complexes to obtain more accurate results and to remove results with a very high similarity score. In the following subsections, the phases of the DPCT method are introduced in detail.

#### Making TAP-Aware Weighted PPI Network

**Figure 2:**
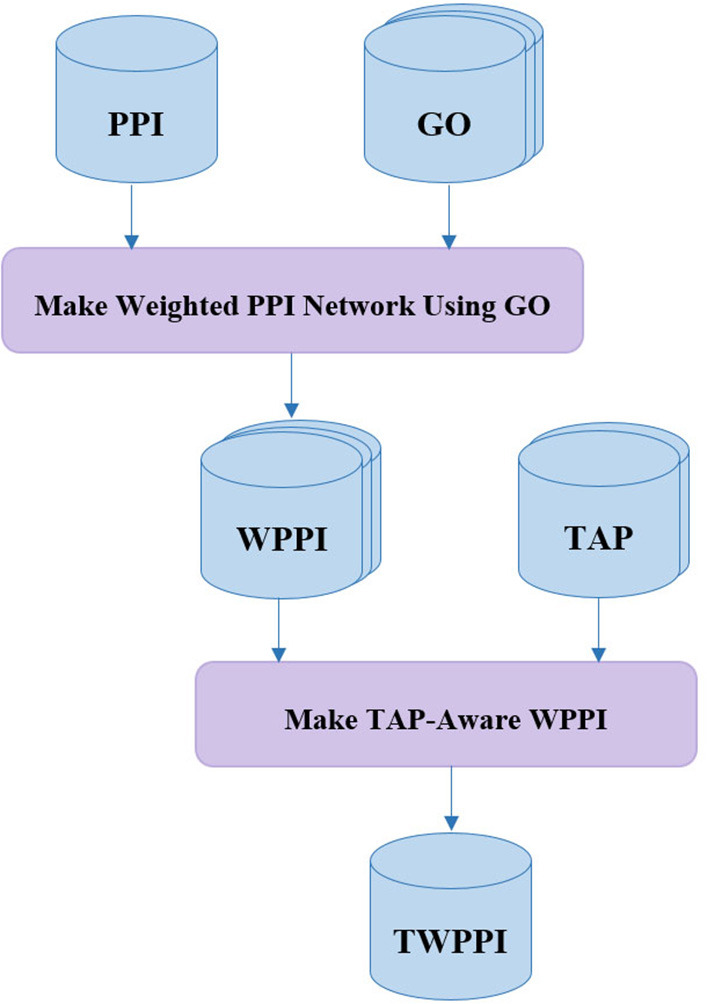
Making TAP-Aware weighted PPI network.

Many state-of-the-art protein complex detection methods use gene ontology (GO) for PPI network noise reduction, i.e., by assigning a weight to each interaction of the PPI network. In DPCT, as shown in Figure 2, TAP data is used as an extra resource, complementary to GO, to allow for more accurate weighted PPI. Both TAP data sets presented by Krogan et al., from LCMS and MALDI are used and normalized between 0 and 1. For each interaction between proteins P_1_ and P_2_ in the PPI network, W_TAP_[P_1_, P_2_] is the weight of the interaction derived from TAP data. W_TAP_[P_1_, P_2_] is calculated by Equation (1); where W_LCMS_[P_1_, P_2_] and W_MALDI_[P_1_, P_2_] are the normalized values of TAP datasets which indicate the purification score between P_1_ and P_2_. If the interaction exists in just one of the TAP sources, W_TAP_[P_1_, P_2_] is set to the available score, and for the interaction that does not exist in both of TAP datasets, W_TAP_[P_1_, P_2_] is set to 0.5 and it does not affect the overall weight of the interaction.

(1) $$\begin{array}{r} W_{TAP}\left[P_{1},\;P_{2}\right]\;=\;\frac{W_{LCMS}\left[P_{1},\;P_{2}\right]\;+\;W_{MALDI}\left[P_{1},\;P_{2}\right]}{2} \end{array}$$

The main source of making a weighted PPI network in DPCT is GO. GO is a dataset with three graphs namely; Biological Process (BP), Cellular Component (CC), and Molecular Function (MF). The GossTo (Caniza et al., 2014) tool and the SimGIC (Pesquita et al., 2008) algorithm are used to make weighted PPI using GO. GossTo is a tool to compute a weight for each interaction of the inputted PPI network based on each graph of the inputted GO dataset. Therefore, there are three weights for each interaction between protein P_1_ and P_2_ in the PPI network based on BP, CC, and MF which we name; W_BP_[P_1_, P_2_], W_CC_[P_1_, P_2_], and W_MF_[P_1_, P_2_]. GossTo can also run a post-processing step including a local search through the graph to increase the accuracy of weighting. Based on Equation (2), W_GO_[P_1_, P_2_] is equal to the average of these three weights. After these steps, W_TAP_[P_1_, P_2_] and W_GO_[P_1_, P_2_] are merged to make the final weight for each interaction among proteins of the PPI network. To make W_TAP_ a coefficient for noise reduction, α is defined by Equation (3).

(2) $$\begin{array}{r} W_{GO}\left[P_{1},\;P_{2}\right]=\;\frac{W_{BP}\left[P_{1},\;P_{2}\right]+\;W_{CC}\left[P_{1},\;P_{2}\right]+\;W_{MF}\left[P_{1},\;P_{2}\right]}{3} \end{array}$$

(3) $$\begin{array}{r} \alpha \left[P_{1},\;P_{2}\right]=1+\left(\left(W_{TAP}\left[P_{1},\;P_{2}\right]-0.5\right)\;\times \;\gamma \right) \end{array}$$

According to Equation (3), α[*P*_1_, *P*_2_] is a value between 1 − γ/2 and 1 + γ/2 based on the W_TAP_[P_1_, P_2_] value for the PPI interaction between P_1_ and P_2_. γ is the impact factor of TAP data, and for a higher value of γ, the final interaction weight will be more affected by W_TAP_. The final weight of interaction between P_1_ and P_2_ is calculated by Equation (4).

(4) $$\begin{array}{r} W\left[P_{1},\;P_{2}\right]\;=\;\alpha \;\times \;W_{GO}\left[P_{1},\;P_{2}\right] \end{array}$$

#### Making Dynamic PPI Subnetworks

**Figure 3:**
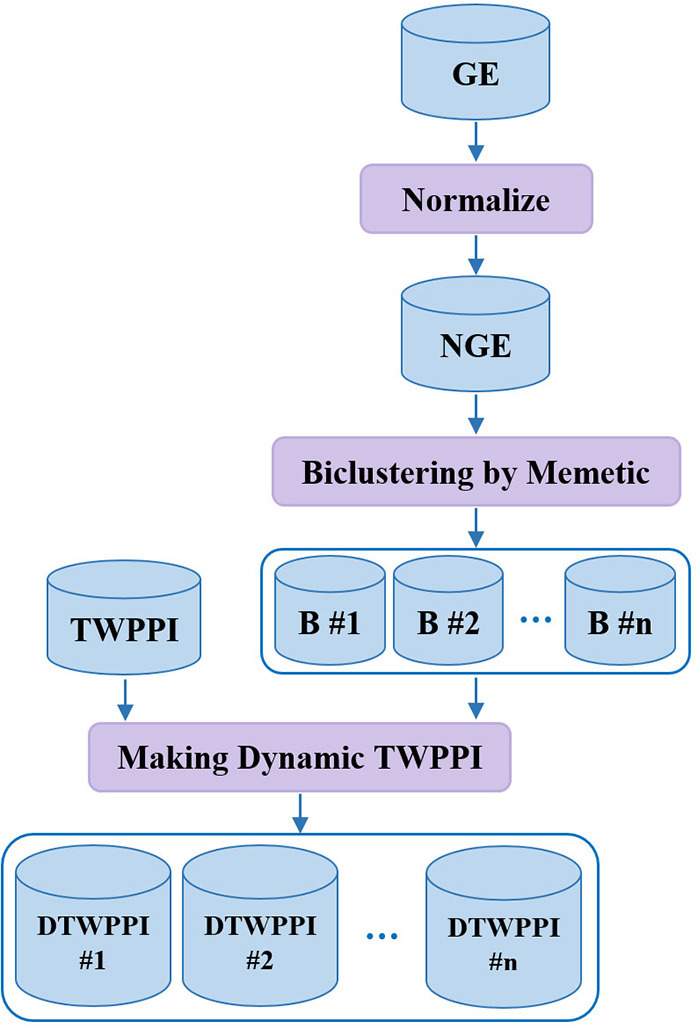
Making dynamic PPI subnetworks.

Recent research demonstrates that cellular systems have a dynamic nature. DPCT tries to take the dynamicity of protein interactions into account to obtain more accurate results (Przytycka et al., 2010). The second phase of DPCT derives dynamic subnetworks from PPI based on gene expression data in three steps, as shown in Figure 3. First, gene expression data is normalized between 0 and 1; next, a memetic algorithm is applied to bicluster the NGE (Normalized Gene Expression) dataset to organize similar active proteins in each bicluster, i.e., PPI subnetworks, and finally, PPI subnetworks are weighted using the TWPPI dataset.

##### GE normalization

To normalize GE, considering GE[m][n] where m is the size of protein array P = {p_1_, p_2_, ., p_m_} and n is the size of time points array T = {t_1_, t_2_, ., t_n_} and GE[i][j] is describing expression level of ith protein over jth time point, for each protein p, according to Equations (5, 6) we first calculate mean (μ) and standard deviation (σ) of values for all time points and then, set its corresponding element of NGE with 0 or 1 based on Equation (7). For each protein in each time point, if the protein's activation is higher than the dynamic threshold [i.e., |μ[*i*] − (σ[*i*] × ε)|], the value will be 1 and otherwise 0. Note that ε is a penalty factor for the normalization process.

(5) $$\begin{array}{r} \mu \left[i\right]=\;\frac{\sum_{j=1}^{n}GE\left[i\right]\left[j\right]}{n} \end{array}$$

(6) $$\begin{array}{r} \sigma \left[i\right]=\sqrt{\frac{\sum_{j=1}^{n}{\left(GE\left[i\right]\left[j\right]-\;\mu \left[i\right]\right)}^{2}}{n}} \end{array}$$

(7) $$\begin{array}{l} \;NGE\left[i\right]\left[j\right]=\{\begin{array}{l} 0,\;\\ \;GE\left[i\right]\left[j\right]\;<\;|\mu \left[i\right]-\left(\sigma \left[i\right]\;\times \;\varepsilon \right)|\\ 1,\;\\ \;GE\left[i\right]\left[j\right]\;\geq \;|\mu \left[i\right]-\left(\sigma \left[i\right]\;\times \;\varepsilon \right)| \end{array} \end{array}$$

##### GE biclustering

DPCT uses a memetic algorithm to bicluster the NGE dataset. The memetic algorithm in its early definition was a modified genetic algorithm with the capability of local refinement by a local-search operator to find a solution for the traveling salesman problem (Norman et al., 1991; Neri and Cotta, 2012). The memetic algorithm was improved and two basic forms of individual learning schemas, named Lamarckian and Baldwinian, were defined (Neri and Cotta, 2012). In Lamarckian learning, any improved individual is forced back into the population to compete for reproduction (Le et al., 2009). Baldwinian learning is another type of memetic algorithm which does not force improved individuals back into the population but updates the fitness of the original individual (Le et al., 2009). In DPCT, a Lamarckian memetic algorithm is used for biclustering discretized gene expression data.

Algorithm 1 describes our memetic algorithm. In line 4, the first population is initialized randomly. Lines 6-15 are the main loop of the memetic algorithm. In line 7, two individuals are selected as parents; in line 8, two children are created by applying a crossover operator on the parents. Line 9 mutate children and in line 10, the fitness of the children is calculated. Line 11 is a local search that tries to optimize children and in line 12, optimized children are inserted into the population. In line 13, two of the worst individuals (i.e., those with least fitness values) are removed from the population. If the end condition is satisfied (i.e., the maximum iteration exceeds or individuals get enough fitness), the loop breaks at line 14. In line 16 the population set is sorted based on fitness values. Afterward, the best solutions are returned as our final GE biclusters in line 17. Below, the encoding of chromosomes, and the operators of our memetic algorithm are described.

**Algorithm 1:**
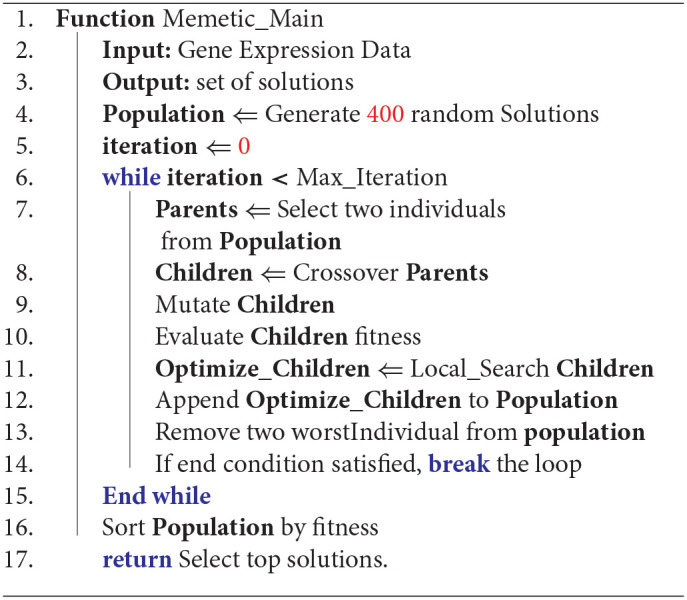
Memetic algorithm

##### Encoding

**Figure 4:**
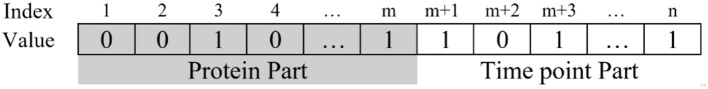
The structure of each chromosome of memetic algorithm.

Each bicluster is an induced matrix of GE (gene expression) so it can be described like B[I][J] such that I ⊆ P and J ⊆ T. For the memetic algorithm, each chromosome maps the structure of a bicluster and is a vector of size m+n genes, where m and n show the number of proteins and time points, respectively. Each gene of the chromosome has a binary value, where 1 and 0 means presence and absence of the corresponding protein or time point in the chromosome. As you can see in Figure 4, in each chromosome, the first m genes are the protein parts describing the presence or absence of each protein, and the time point part starts from m+1 to m+n genes, describing the presence or absence of each time point.

##### Fitness function

The goodness of a bicluster depends on the number of its active proteins. We used a memetic algorithm to search for a group of proteins and time points i.e., a bicluster such that most selected proteins are active in the selected time points. Equation (8) is used to measure the quality of each detected bicluster. As mentioned above, a bicluster is a two-dimensional matrix, like B[I][J] such that I is a subset of P and J is a submatrix of T. For each selected protein, we have |J| time points, so we have |I| ^*^ |J| expression values and we expect most of them to be active. To calculate F_b_, we find the proportion of active to inactive expressions. In the best case, if all selected proteins are active in all selected time points, F_b_ becomes equal to 1 and in the worst case, if there is no active protein among all selected proteins and time points, F_b_ will be 0.

(8) $$\begin{array}{r} F_{b}=\;\frac{\;\sum_{i\in I}\sum_{j\in J}\left(NGE\left[i\right]\left[j\right]\;==\;1\right)}{\left|I|\times |J\right|} \end{array}$$

##### Selection and crossover

In the proposed memetic algorithm, a binary tournament is used to select two best individuals for crossover. Two points crossover is used, and crossover is only applied to the protein part of the chromosome, so the time point part will not be affected. Having two selected individuals, two crossover points on the protein part of each individual are selected randomly and the part of parent chromosomes between selected points will be swapped with the probability of 0.9; as a result, we have two new individuals.

##### Mutation

A mutation operator with a different probability is used for the protein part and time point part. For the protein part, each cell of the chromosome will be flipped with the probability of 0.001 and in the time point part, each cell will be flipped with the probability of 0.1. As described above, crossover did not apply to the time point part so we increased the probability of mutation for the time point part to allow the memetic algorithm to walk through different situations and to find better solutions.

##### Local search

Local search of the memetic algorithm is applied to any new individual added to the population. In local search, each bit of the chromosome is selected and flipped with the probability of 0.8. If the fitness of the derived chromosome is not increased, the selected bit is reversed to its previous value, otherwise, the new value is saved for the bit in the chromosome. Then the procedure will continue for the next bit.

##### Making a dynamic subnetwork

After normalizing gene expression data and finding biclusters using a memetic algorithm, the third step is to extract dynamic subnetworks from PPI correspondent to the detected gene biclusters. For each bicluster B_i_, we make a correspondent dynamic subnetwork of PPI, DTWPPI#i that contains only active proteins which are members of bicluster B_i_ and their interactions.

#### Detecting Protein Complexes From Dynamic Subnetworks

In section Making Dynamic PPI Subnetworks, the static PPI network is divided into some DTWPPI (Dynamic TAP-Aware Weighted PPI) that has the most active proteins at one or more than one time points. These DTWPPIs may overlap due to the nature of biclustering. We apply the CAMWI (Lakizadeh et al., 2015a) method to detect protein complexes on each DTWPPI. The CAMWI method detects protein complexes from a weighted PPI network in four steps. The first step calculates the weighted local clustering coefficient for each protein of a PPI subnetwork and selects seeds from them. For each seed, a core is created by attaching some proteins of the DTWPPI subnetwork to seeds that have the highest interactions among themselves and the seed. The third step of CAMWI extends each core by selecting another set of proteins from the DTWPPI subnetwork, called attachments, and attaches them to its cores to make a protein complex. Attachments are selected from the direct neighborhood of the corresponding core of the DTWPPI subnetwork. Choosing a protein as an attachment depends on the count of its interactions with the core and a threshold parameter β. The last step is to filter all detected protein complexes and remove redundant protein complexes. The result of this stage of DPCT is a set of protein complexes detected from the set of DTWPPIs.

#### Analysis and Aggregation

In section Detecting Protein Complexes From Dynamic Subnetworks CAMWI is applied to dynamic PPI subnetworks to detect protein complexes so the result is a Z set of protein complexes. The detected bicluster may overlap and this overlapping will propagate to DTWPPI_*i*_ so we may have a protein complex completely or partially in more than one set of results. In this section, we analyze the derived protein complex sets and make a final set of protein complexes using a four step algorithm. First, we need the similarity score of two protein complexes; The Jaccard index (Srihari and Leong, 2014) is used to calculate the similarity score of two protein complexes. If C1 and C2 are two protein complexes, J(C1, C2) can be calculated by Equation (9) where, |C1 ∩ C2| is the count of mutual proteins in C1 and C2, and |C1 ∪ C2| is the size of the set that contains all proteins of C1 and C2.

(9) $$\begin{array}{r} J\left(C1,\;C2\right)=\;\frac{\left|C1\;\cap \;C2\right|}{\left|C1\;\cup \;C2\right|} \end{array}$$

Algorithm 2, presents the analysis and aggregation phase. In the first step, G, a global set of protein complexes is defined. G contains all detected protein complexes from all result sets of CAMWI for each DTWPPI. All complexes of G with a count of proteins less than three are removed. For another complex in G, if the count of proteins is less than six, the complex is added to the final result directly and complexes with a size of 5 or more, will be categorized by similarity score to some set, so that all complexes with a similarity score >0.8 are categorized to a set. The result of phase 1 is that all complexes in some category of protein complexes have a similarity score >0.8. This part of the algorithm is shown in lines 6–13 of Algorithm 2.

**Algorithm 2:**
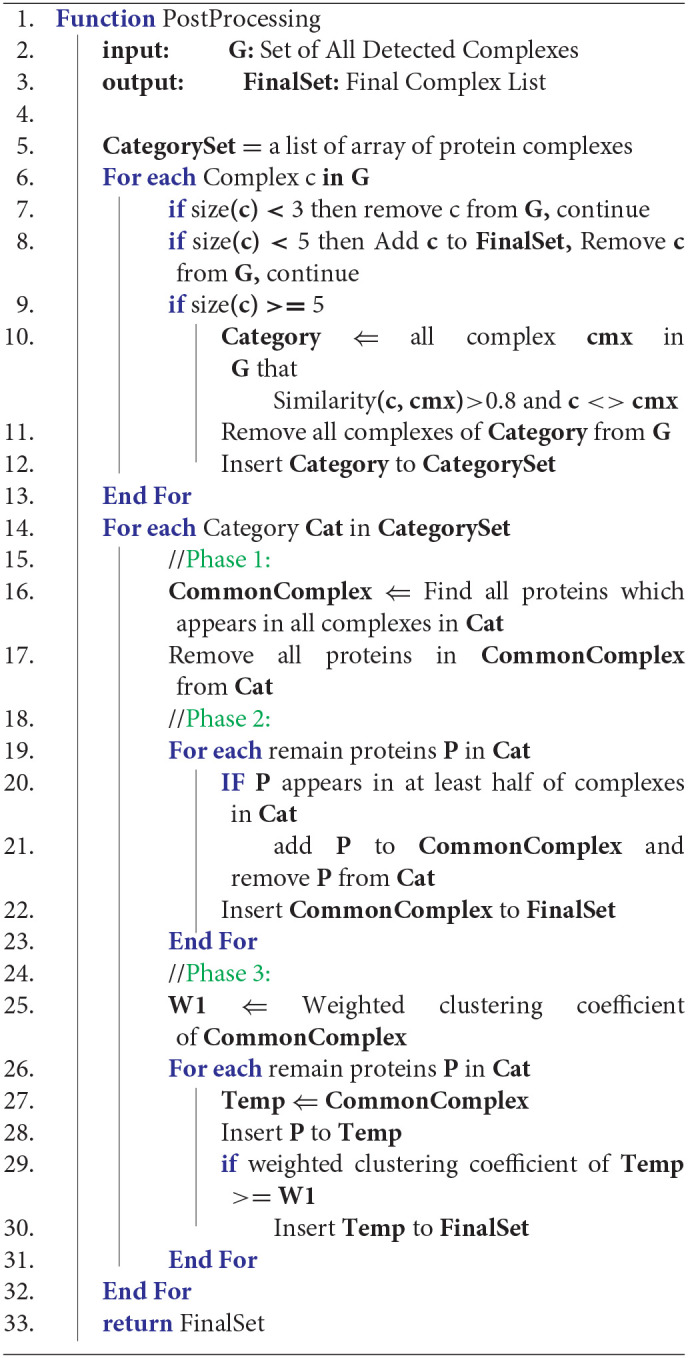
Analysis and aggregation

Lines 14-32 of Algorithm 2 describe the main loop of the post-processing algorithm which contains three phases. Phase 1, 2, and 3 will be applied to each category separately. In the first phase, shown in lines 16 and 17, the common part of all protein complexes from each category is found (CommonPart) and is considered a protein complex. All proteins of the CommonPart are removed from all protein complexes in the corresponding category. In the next phase, in lines 19–23 of Algorithm 2, each protein in each category is checked and if it participates in at least half of the protein complexes in the corresponding category, it is appended to the CommonPart of the category and the protein is removed from all protein complexes in the corresponding category. In this stage, the CommonPart is added to the final result.

Remaining proteins in each category will enter the last phase of analysis and aggregation algorithm which is defined in lines 25–31. In this phase, each remaining protein will be added to a copy of CommonPart and if the new complex has a greater weighted clustering coefficient (WCC) (Kalna and Higham, 2007) than before, the new complex will be added to the final result, otherwise, the protein will be ignored. Line 25 calculates WCC for the common complex and in lines 27–30, each protein is added to the common part and its new WCC is compared with its original one to decide whether to create a new protein complex or to ignore the protein. The weighted clustering coefficient for a protein complex can be calculated by Equations (10, 11). Considering that C(V,E) is a protein complex with V = {v_1_, v_2_, …, v_m_} proteins and E = {e_1_, e_2_, …, e_n_} edges among proteins; WCC(v) for each protein can be determined by Equation (10). Where W(e) demonstrates the weight of edge e, and L is the set of all edges of v. WCC(C) for each protein complex C(V, E) is computed by Equation (11). At the end of the 4th phase, all categories must be empty, and the final set contains the detected protein complexes by DPCT.

(10) $$\begin{array}{r} WCC\left(v\right)=\;\frac{\sum_{e\in L}W\left(e\right)\;}{|L|\;\times \left(|L|-1\right)} \end{array}$$

(11) $$\begin{array}{r} WCC\left(C\right)=\;\frac{\sum_{v\in V}WCC\left(v\right)}{\left|V\right|} \end{array}$$

## Experiments and Results

### Evaluation Measures

To assess the quality of the proposed DPCT method, we use precision, recall and F-1 measures which are the common measurements for protein complex detection methods. The Jaccard index, defined in section Analysis and Aggregation, is used to specify the overlap score between the detected protein complex and the benchmark complex. B = {b_1_, b_2_, …, b_n_ } denotes the benchmark complex set and C = { c_1_, c_2_, ., c_m_ } denotes the set of detected complexes by DPCT, then as mentioned above, J(b_i_, c_i_) can be determined by Equation (9) and if *J*(*b*_*i*_, *c*_*i*_)≥*th* then c_i_ is considered as a true detected complex. In this study, like other state-of-the-art articles, we set *th* to 0.25. The precision measure represents how much of the detected complexes are matched correctly with the benchmark and the recall measure represents how much of the real complexes are detected using the DPCT method. The F-1 measure is the harmonic mean of precision and recall and can be used to assess the overall performance of detection methods. Precision, recall, and F-1 measures are defined in Equations (12–14) (Li et al., 2010).

(12) $$\begin{array}{r} Precision=\;\frac{\left|\left\{c_{i}\;|\;c_{i}\in C\;\wedge \;\exists \;b_{i}\in B\;:J\left(c_{i},\;b_{i}\right)\geq th\right\}\right|}{\left|C\right|} \end{array}$$

(13) $$\begin{array}{r} Recall=\;\frac{\left|\left\{b_{i}\;|\;b_{i}\in B\;\wedge \;\exists \;c_{i}\in C\;:J\left(b_{i},\;c_{i}\right)\geq th\right\}\right|}{\left|B\right|} \end{array}$$

(14) $$\begin{array}{r} F-Measure=\;\frac{2\;\times Precision\;\times Recall}{Precision+Recall} \end{array}$$

### DPCT Parameter Tuning

DPCT uses five parameters in the process of detecting protein complexes. In a real-world situation, it may be hard to tune these parameters but in this section, we analyze the effect of each parameter on the final result. It can help us obtain an approximate estimation for each parameter and this analysis shows how to decline the range of parameters. Furthermore, there are many protein complex datasets presented by biologists and we can use some set of known protein complexes to test and tune the parameters of DPCT.

#### Tuning **α** and **β** Parameters

**Figure 5:**
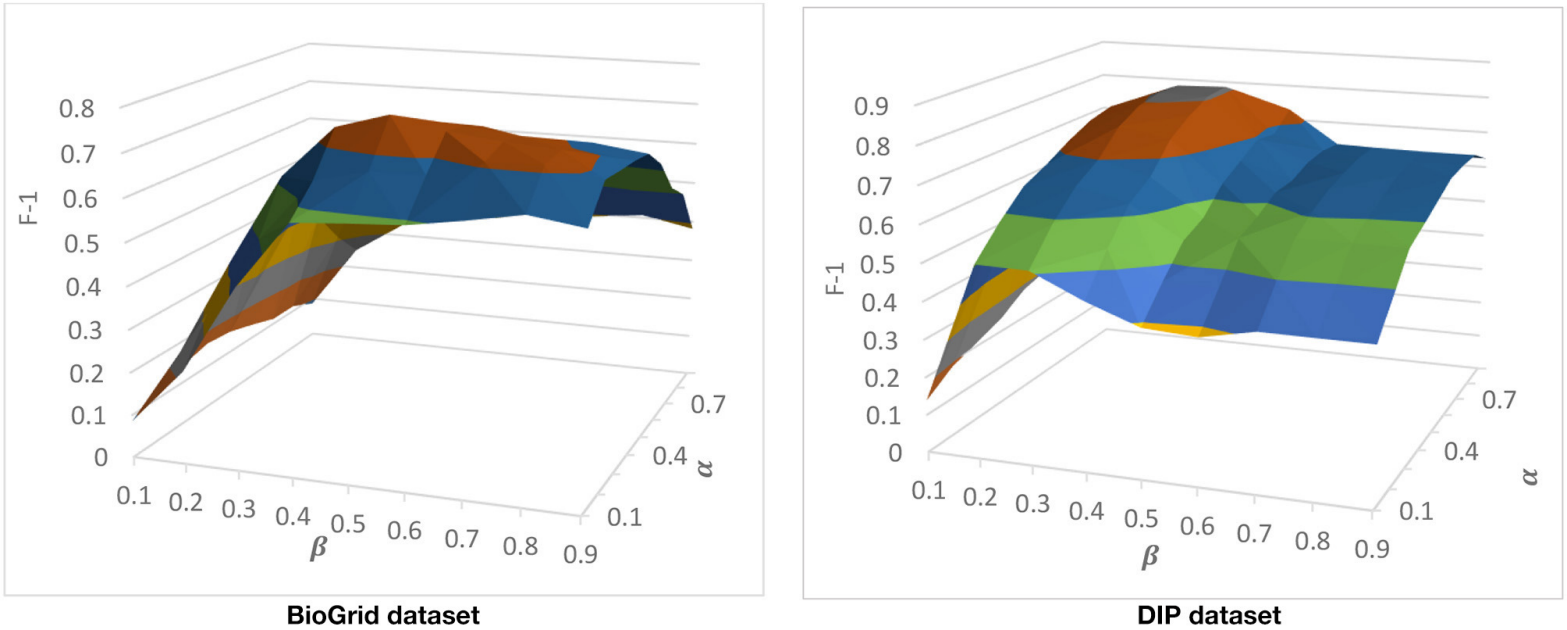
Tuning α and β with CYC2008 benchmark.

There are two parameters that come from CAMWI. CAMWI uses α and β to tune the seed generation step and the core growing step. These parameters and CAMWI, are used in the 3rd phase of DPCT to detect protein complexes from dynamic PPI subnetworks. CAMWI, with lower values of α finds more seeds and consequently more protein complexes. β is a threshold in the process of selecting proteins as attachments to a core, so greater values of β will decrease the size of detected protein complexes. To find the best values for α and β for each dataset, we run DPCT with α and β in range of [0.1 . 0.9]. Figure 5 shows the F-1 Measure for different values of α and β with the CYC2008 benchmark for BioGrid and DIP datasets. According to the results, for the BioGrid dataset the best result was achieved when α was set to 0.3 and β was set to 0.5. Furthermore, for the DIP dataset, α = 0.55 and β = 0.7, which causes a higher F-1 Measure.

#### Tuning the **γ** Parameter

In the first phase of DPCT, TAP, and GO datasets are used to make a weighted PPI network. Since, we face a high degree of missing data in TAP, GO is considered as the main source of weighting and TAP data is considered to be complementary. According to Equation (3), the efficacy of TAP on GO is controlled by γ. γ can be set from [0 . 1] and for γ = 1; the GO score will be multiplied by a factor between 0.5 and 1.5 based on the TAP score. We can set γ based on the availability and the missing rate of TAP data and the reliability of GO data.

**Figure 6:**
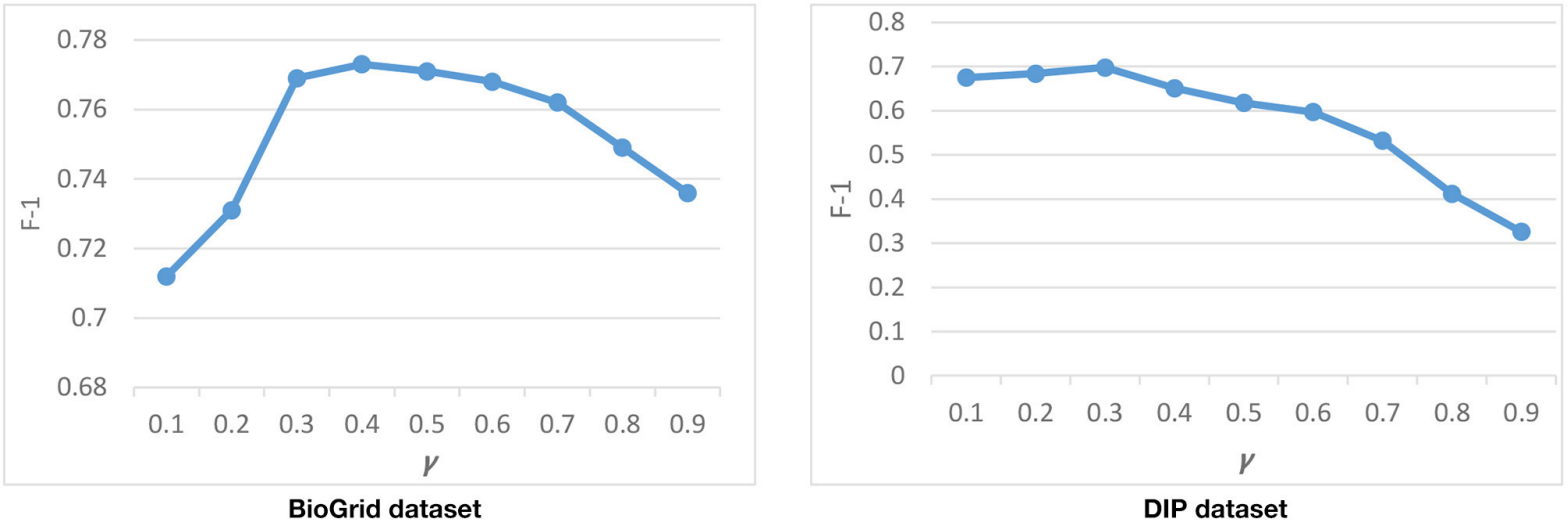
Tuning γ with CYC2008 benchmark.

To find a good value for γ, we run DPCT with different values of γ. Figure 6 shows the result of DPCT with γ in range of 0.1–0.9 for the CYC2008 benchmark in BioGrid and DIP datasets. Considering Figure 6, the best γ for BioGrid and DIP datasets are 0.4 and 0.3, respectively. These values of γ causes GO to be multiplied by a number in a range of [0.8, 1.2] and [0.85, 1.15] for BioGrid and DIP datasets based on TAP data, respectively.

#### Tuning the **ε** Parameter

**Figure 7:**
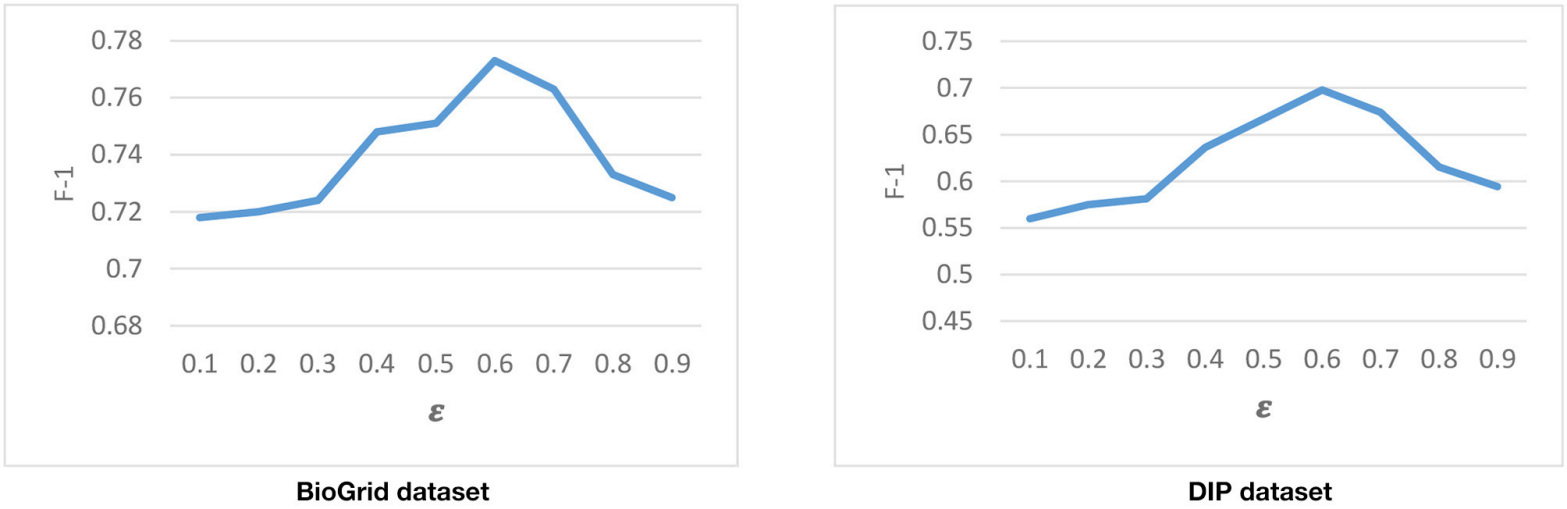
Tuning ε with CYC2008 benchmark.

Another parameter that is used in the second phase of DPCT is ε, our threshold to discretize gene expression data. So for the same gene expression data, ε is supposed not to change for different PPI networks. Considering Equation (7), higher values of ε lead to having more inactive genes and it increases the sparseness of the gene expression matrix and *vice versa*. Figure 7 represents the result of running DPCT for possible values of ε for the BioGrid and DIP dataset with the CYC2008 benchmark. We can see that the best value of ε for both BioGrid and DIP is 0.6.

#### Tuning the Parameter

**Figure 8:**
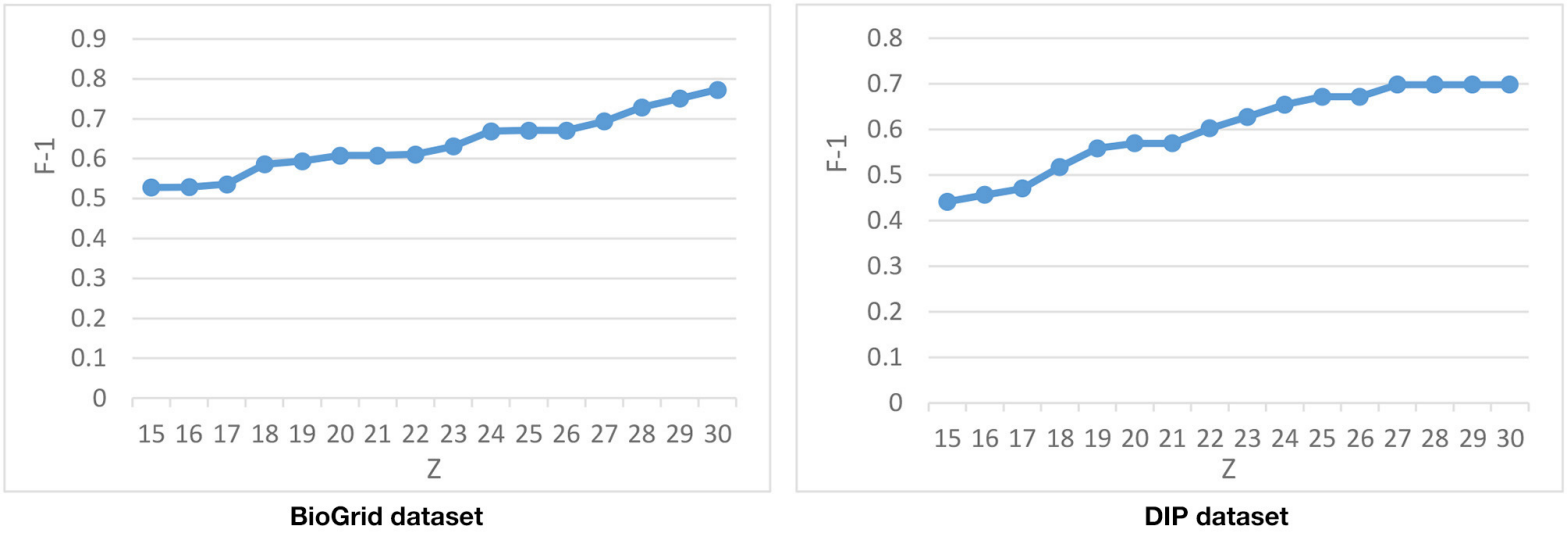
Tuning with CYC2008 benchmark.

The last parameter that is used in the second phase of DPCT is, which declares the number of derived biclusters and, consequently, the number of dynamic PPI subnetworks. Figure 8 represents the F-1 Measure of DPCT when changes in the range of 15–30 in the BioGrid and DIP datasets with the CYC2008 benchmark. Due to Figure 8, the best value for in BioGrid and DIP is 30 and 27, respectively. Experiments show that increasing has no effect on the result of DPCT because the analysis and aggregation phase will merge or remove the redundant detected protein complexes.

### Best Results and Comparison With Other Methods

We applied DPCT on BioGrid and DIP datasets as input PPI networks and used CYC2008 and MIPS as benchmarks to confirm the correctness of detected protein complexes. We ran DPCT on a computer with 2.5 GHz Intel CoreI7 CPU and 4 GB of RAM and it took about 12 s on average. We also ran BiCAMWI and CAMWI on the same machine and they required 25 and 3.5 s to finish, respectively. Based on reports, COACH takes <40 s on a 3.5 GHz CPU and 3 GB of RAM to generate results, and TINCD takes between 785 and 4,200 s to conclude its calculation on a system with a 2 × 2.1 GHz CPU and 12 GB RAM.

Table 1 shows the best result of the DPCT and some other state-of-the-art methods to detect protein complexes in the BioGrid Dataset. In this test, we use the best values for each parameter to obtain the best accuracy. Based on Table 1, in most cases, the DPCT detects protein complexes more accurately than other methods. In the BioGrid dataset and MIPS benchmark, EWCA has the best recall with ~2% better results than DPCT. Table 2 presents the best result of the DPCT and other methods on the DIP dataset. With respect to precision, the DPCT does not have the best value in the DIP dataset and BiCAMWI is 6.4 and 8.3% better than our method for CYC2008 and MIPS benchmarks; but in recall, the DPCT is 35 and 19.4% better than BiCAMWI for CYC2008 and MIPS benchmarks. Overall, F-1 Measure values of the DPCT are better than all other methods. Note that in Tables 1, 2, the best result of all methods is shown and for the DPCT, the tuned values of parameters described in section DPCT Parameter Tuning are used. For some methods like InteHC, IFPA, and ONCQS, there were differences between the datasets, benchmarks, or evaluation metrics; so, we only report the comparable results. The results for InteHC are reported from Ou-Yang et al. (2016b).

**Table 1:** Comparison of the proposed DPCT method with other methods in terms of Precision, Recall, and F-1 measures on BioGrid dataset.

**Benchmark**	**CYC2008**			**MIPS**		
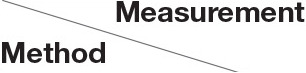	**Precision**	**Recall**	**F-1**	**Precision**	**Recall**	**F-1**
Proposed DPCT	**0.693**	**0.875**	**0.773**	**0.608**	0.738	**0.667**
BiCAMWI (Lakizadeh and Jalili, 2016)	0.443	0.695	0.534	0.412	0.58	0.481
TS-OCD (Ou-Yang et al., 2014)	0.363	0.741	0.478	0.312	0.575	0.404
CAMWI (Lakizadeh et al., 2015a)	0.4	0.61	0.5	0.29	0.5	0.36
Cluster-ONE (Nepusz et al., 2012)	0.312	0.655	0.422	0.208	0.445	0.283
PCD-GED (Lakizadeh et al., 2015b)	0.43	0.63	0.51	0.35	0.53	0.42
COACH (Wu et al., 2009)	0.284	0.716	0.406	0.221	0.562	0.317
GMFTP (Zhang et al., 2014)	0.291	0.783	0.424	0.283	0.753	0.411
EWCA (Wang and Caixia Wang, 2019)	0.579	0.809	0.675	0.582	**0.756**	0.657
InteHC (Wu et al., 2013)	0.213	0.527	0.303	N/A	N/A	N/A

*Best values are bolded*.

**Table 2:** Comparison of the proposed DPCT method with other methods in terms of Precision, Recall, and F-1 measures on DIP dataset.

**Benchmark**	**CYC2008**			**MIPS**		
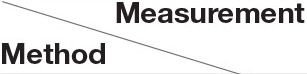	**Precision**	**Recall**	**F-1**	**Precision**	**Recall**	**F-1**
Proposed DPCT	0.557	**0.883**	**0.698**	0.524	**0.708**	**0.602**
BiCAMWI (Lakizadeh and Jalili, 2016)	0.621	0.533	0.585	**0.607**	0.514	0.556
TS-OCD (Ou-Yang et al., 2014)	0.429	0.524	0.472	0.397	0.449	0.421
CAMWI (Lakizadeh et al., 2015a)	0.43	0.47	0.45	0.35	0.5	0.411
Cluster-ONE (Nepusz et al., 2012)	0.301	0.447	0.36	0.247	0.331	0.283
PCD-GED (Lakizadeh et al., 2015b)	0.485	0.52	0.5	0.45	0.44	0.444
COACH (Wu et al., 2009)	0.295	0.553	0.385	0.269	0.5	0.35
GMFTP (Zhang et al., 2014)	0.266	0.665	0.38	0.275	0.698	0.395
EWCA (Wang and Caixia Wang, 2019)	0.523	0.707	0.602	0.499	0.701	0.583
IFPA (Lei et al., 2019)	**0.694**	0.461	0.554	N/A	N/A	N/A
ONCQS (Zhao and Lei, 2019)	0.356	0.826	0.497	N/A	N/A	N/A

*Best values are bolded*.

## Analytical Discussion

### The Effectiveness of TAP Data

**Figure 9:**
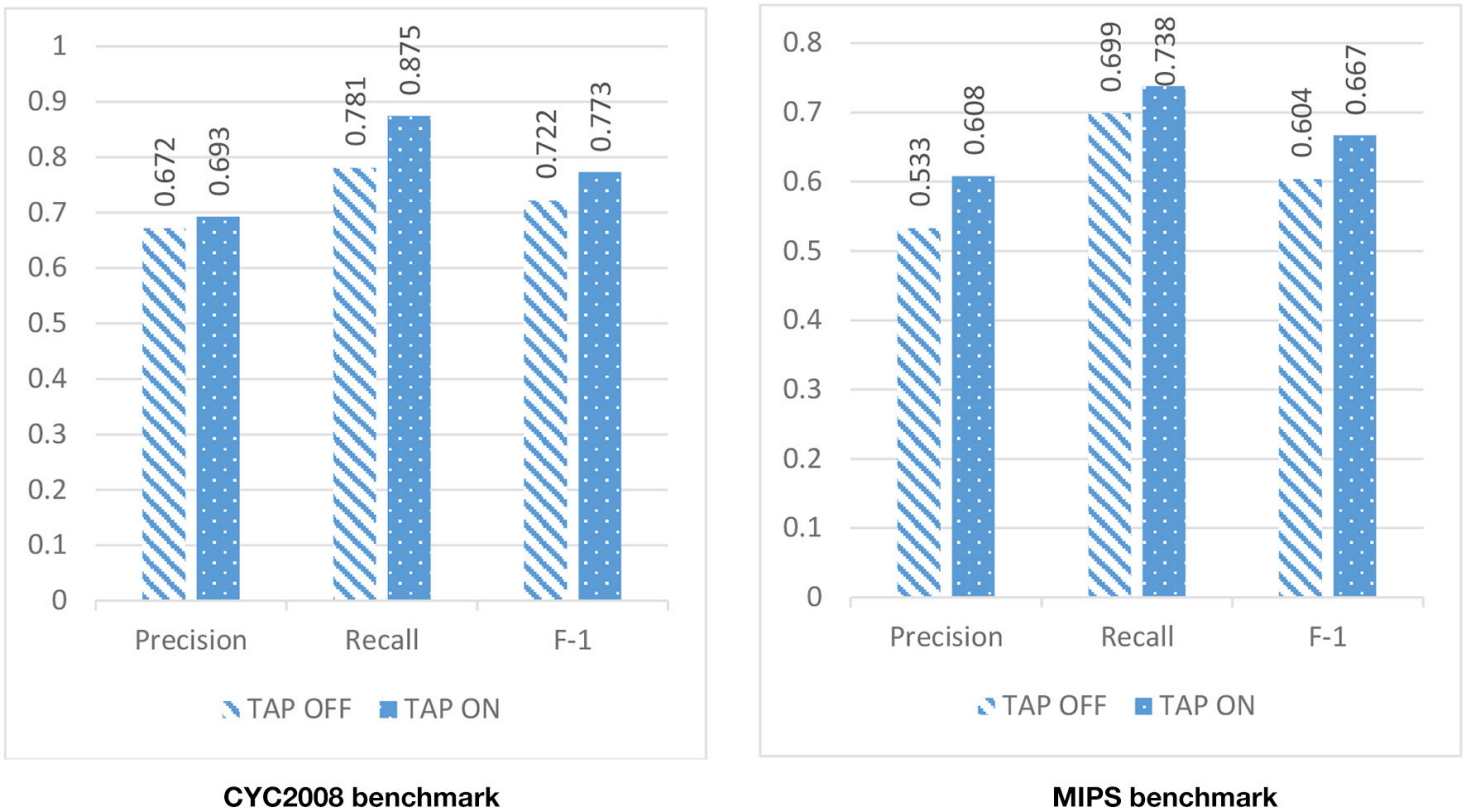
The effectiveness of DPCT when TAP data is off/on in BioGrid dataset with CYC2008 and MIPS benchmarks.

**Figure 10:**
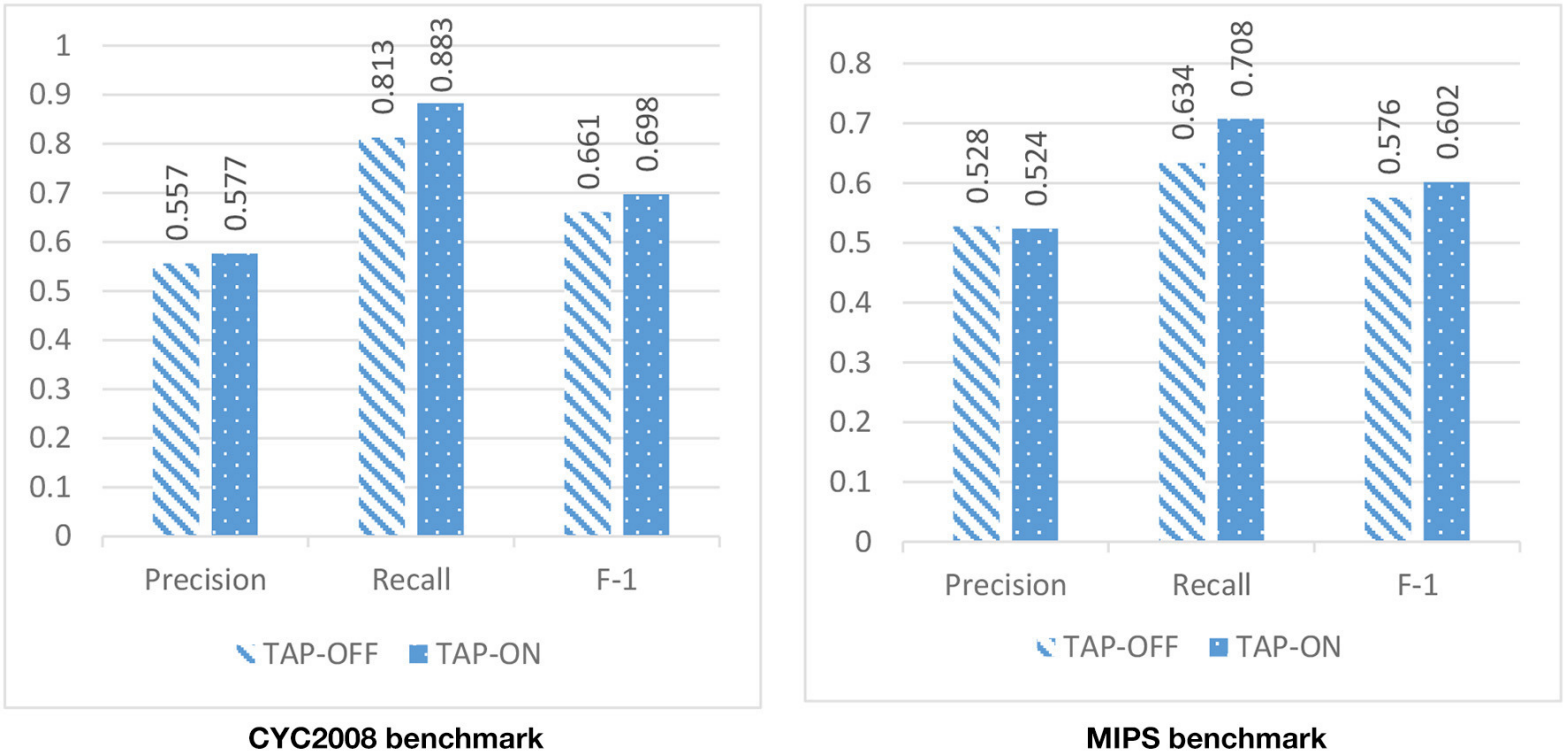
The effectiveness of DPCT when TAP data is off/on in DIP dataset with CYC2008 and MIPS benchmarks.

Due to the high level of noise in the PPI network, DPCT used GO and TAP data to assign a weight to each interaction of the PPI network. To assess the effect of using TAP in the accuracy of detected protein complexes, we ran DPCT without considering TAP data and we compared its results with normal the DPCT method which uses both GO and TAP data. In the TAP-OFF situation, a weighted PPI network was created with GO data only. Figures 9, 10 show the results of DPCT in TAP-OFF and TAP-ON (normal) mode for BioGrid and DIP datasets with CYC2008 and MIPS benchmarks, respectively. Considering Figure 9, we realize that by taking TAP data into account, the quality of DPCT is enhanced. In TAP-ON mode for BioGrid data, the F-1 Metric increases ~5% for the CYC2008 benchmark and 6% for the MIPS benchmark. According to Figure 10 in the DIP dataset, in TAP-ON mode, the Recall metric increases about 7% for CYC2008 and MIPS benchmarks in comparison with the TAP-OFF mode. This experiment shows that using both TAP and GO removes more noise from PPI networks. A comparison between DPCT and other methods (Wu et al., 2013; Ou-Yang et al., 2016a,b) that use only GO to make weighted PPI, also represents a positive effect of TAP data.

### The Effectiveness of the Memetic Algorithm

**Figure 11:**
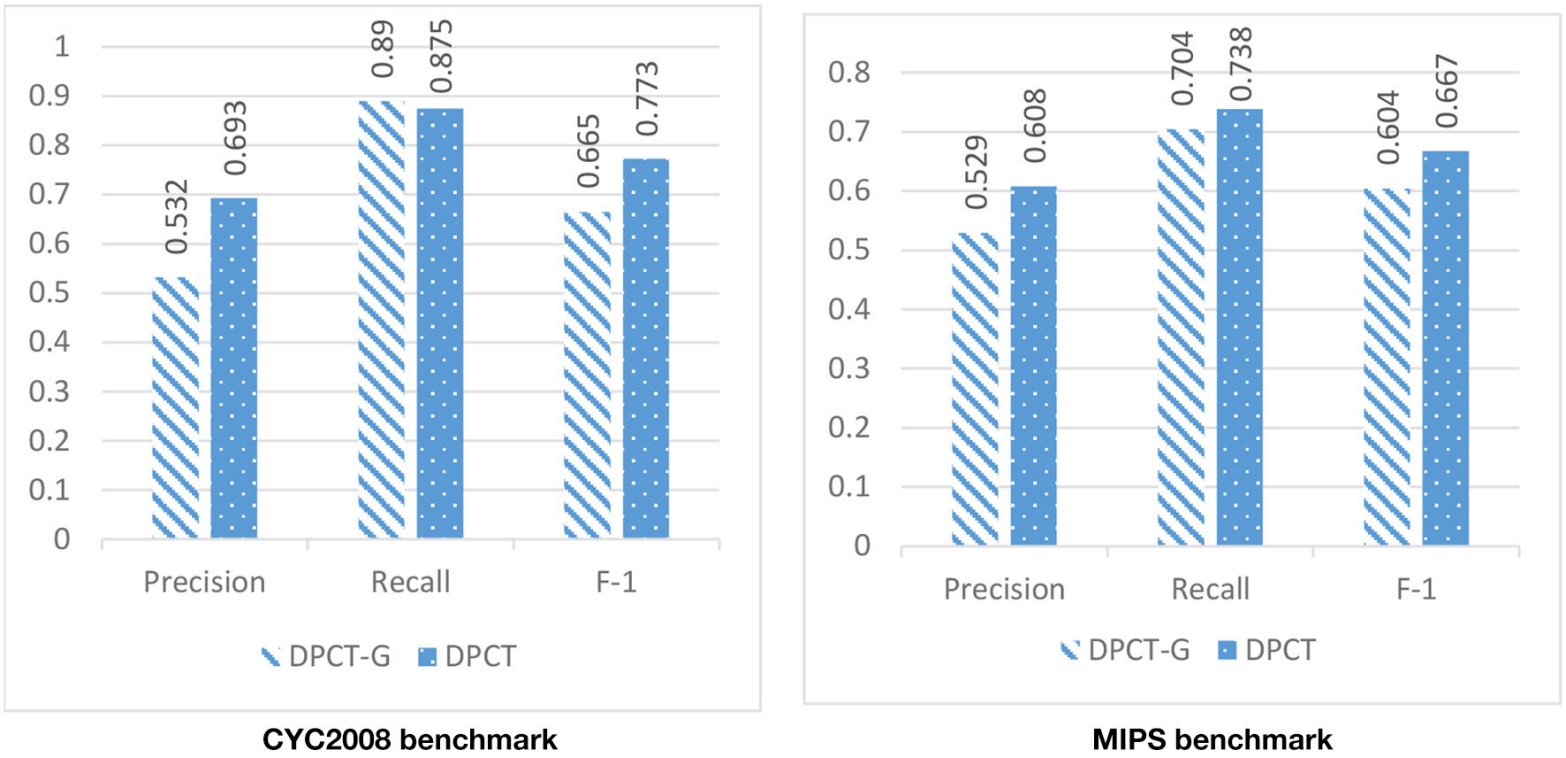
The effectiveness of using the novel memetic algorithm (in DPCT) in compare with using GA-DCT in DPCT (DPCT-G) in BioGrid dataset with CYC2008 and MIPS benchmarks.

**Figure 12:**
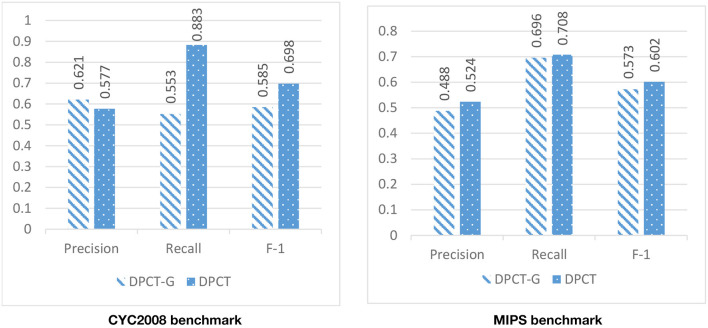
The effectiveness of using the novel memetic algorithm (in DPCT) in compare with using GA-DCT in DPCT (DPCT-G) in DIP dataset with CYC2008 and MIPS benchmarks.

In DPCT, a memetic algorithm is proposed to bicluster gene expression data and to make dynamic PPI subnetworks. Some state-of-the-art methods for protein complex detection, use metaheuristic algorithms to bicluster gene expression data. BiCAMWI is a method that defines and uses GA-DCT, a novel genetic algorithm for clustering gene expression data. To investigate the effect of our proposed memetic algorithm, we used GA-DCT instead of our proposed memetic algorithm. Figures 11, 12 compare the results of DPCT and DPCT-G (DPCT with GA-DCT Genetic algorithm) for BioGrid and DIP datasets. We can see that DPCT with the proposed memetic algorithm has a better F-1 value in all datasets and benchmarks. Note that, for the CYC2008 benchmark, DPCT has a lower recall in the BioGrid dataset and a lower precision in the DIP dataset. The main difference between the proposed memetic algorithm and GA-DCT is in the selection, cross over, mutation, and the fitness function of the algorithm. The new definition of the fitness function allows the algorithm to choose promising individuals. Moreover, applying the local search in memetic algorithm decreases the running time of the algorithm and increases the performance of final solutions.

### The Effectiveness Analysis and Aggregation Phase

The last phase of the DPCT method is analysis and aggregation, a post-processing phase. After detecting protein complexes from each dynamic PPI subnetwork, due to the nature of biclustering, there may be many redundant and highly similar protein complexes in the result set. In this post-processing phase, all highly similar protein complexes are aggregated and also, we can control and decrease the negative effect of the parameter which controls the number of dynamic PPI subnetworks. By increasing the value of, the total number of dynamic subnetworks will grow up; therefore, the total number of redundant or highly similar detected protein complexes are increased. In this situation, the analysis and aggregation phase remove or merge redundant protein complexes.

In order to assess the effect of the post-processing phase, we ran DPCT without its last phase. Table 3 shows the result of detected protein complexes in PostProcessing-On and PostProcessing-Off modes. We can see that the post-processing phase reduced the number of redundant protein complexes. The analysis and aggregation phase are also evaluated based on precision, recall, and F-1 measures. In the BioGrid dataset with the CYC2008 benchmark, when DPCT uses the post-processing phase we see a 0.9% fall in precision and no change in recall, so, DPCT without the analysis and aggregation phase has a 0.6% better F-1 measure. However, for the MIPS benchmark, DPCT with the analysis and aggregation phase has a 3.4, 3.3, and 3.9% rise in precision, recall, and F-1 measure, respectively.

**Table 3:** The number of detected protein complexes with and without post-processing phase of DPCT (analysis and aggregation) for BioGrid and DIP datasets with both CYC2008 and MIPS benchmarks.

**Benchmark**	**CYC2008**		**MIPS**	
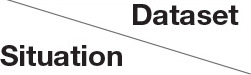	**BioGrid**	**DIP**	**BioGrid**	**DIP**
PostProcessing-OFF	250	145	248	141
PostProcessing-ON	181	138	162	138

In the DIP dataset with the CYC2008 benchmark, when DPCT uses the post-processing phase, we see a rise in precision and F-1 measures by 1.4 and 0.9% respectively. Moreover, for the MIPS benchmark, DPCT with the analysis and aggregation phase, gain the rise of precision, recall, and F-1 Measures by 2.6, 2.6, and 2.7% respectively. As the results show, the last phase of DPCT, not only removes redundant detected protein complexes, so decreases the size of the result set, but also in most cases, it leverages the quality of results and removes incorrectly detected protein complexes.

## Conclusion

Protein complexes play an important role in the cellular system. Many researchers therefore work on developing new methods to detect protein complexes. There are many methods that use different approaches like clustering, seed generation, and core-attachment to detect protein complexes. In recent years, many methods first make a dynamic PPI network and then detect protein complexes from the network.

In this paper, we present DPCT, a novel method to detect protein complexes. DPCT uses TAP data as minor data, and as the complement of GO to make a more accurate weighted PPI network. An extended memetic algorithm is used to bicluster gene expression data and to consequently make a dynamic PPI subnetwork. After applying the third phase of DPCT on each dynamic PPI subnetwork, detected protein complexes are aggregated so that redundant detected protein complexes are removed from the final result set. A comparison between DPCT and other state-of-the-art methods shows that DPCT can detect protein complexes with better values of precision, recall, and F-1 measures.

For future works, we suggest using a learner to fuse the weights that come from TAP and GO to obtain a better weighted PPI network. Gene expression is experimental data that may have a high noise rate so any effort to decrease the noise rate of gene expression data can increase the quality of the detection method. Using other methods to make dynamic PPI subnetworks that are more consistent with biological concepts, such as other evolutionary algorithms, can be helpful in increasing the accuracy of protein complex detection.

## Data Availability Statement

The datasets used in this study can be found at https://github.com/alisn72/DPCT.

## Author Contributions

AS was responsible for development phase of the main algorithm and also draft the article. SJ also revised the drafted article and approved the content to publish the paper. All authors were responsible for designing the algorithm.

## Conflict of Interest

The authors declare that the research was conducted in the absence of any commercial or financial relationships that could be construed as a potential conflict of interest.
